# Utilizing GIS to Reduce Child Mortality: Strategic Policy Actions for Health Equity

**DOI:** 10.34172/hpp.45251

**Published:** 2026-08-21

**Authors:** Tahereh Samimi, Aynaz Nourani, Toomas Timpka, Bahlol Rahimi

**Affiliations:** ^1^Student Research Committee, Urmia University of Medical Sciences, Urmia, Iran; ^2^Health and Biomedical Informatics Research Center, Urmia University of Medical Sciences, Urmia, Iran; ^3^Department of Medical Informatics, School of Allied Medical Sciences, Urmia University of Medical Sciences, Urmia, Iran; ^4^Department of Health, Medicine and Caring Sciences, Division of Society and Health, Linköping University, Linköping, Sweden

**Keywords:** Child mortality, Geographic information system, Health equity, Policy brief

## Abstract

Under-five mortality remains a critical issue with persistent geographic disparities. While Geographic Information Systems (GIS) can pinpoint mortality hotspots and local risk factors, its integration into healthcare systems is often fragmented due to inadequate infrastructure, isolated data systems, and insufficient technical expertise. This policy brief outlines five integrated recommendations: investing in sustainable GIS infrastructure and governance, adopting advanced interactive technologies, improving data interoperability, fostering interdisciplinary collaboration and capacity development, and enhancing community involvement with an equity focus. These measures would transform GIS from a basic mapping tool into a dynamic decision-support system. By integrating facility-based reports, community health worker data, and demographic surveillance, this system enables near-real-time identification of mortality hotspots. Consequently, it optimizes resource allocation and supports culturally appropriate, equity-driven interventions to effectively address geographic inequalities in child survival.

## Introduction

 Global efforts under Sustainable Development Goal (SDG) 3.2 focus on reducing child mortality. However, health strategies in many low- and middle-income countries (LMICs) often fail to adequately address subnational mortality hotspots, which are geographic regions where preventable child deaths remain highly concentrated. In 2022, an estimated 4.9 million children under five years of age died, and 78 percent of these deaths occurred in sub-Saharan Africa and South Asia.^1^ While the global under-five mortality rate declined by 61 percent between 1990 and 2020, decreasing from 93 to 37 deaths per 1,000 live births, the burden remains substantial in LMICs.^2^

 This persistent burden is shaped by the combined effects of limited access to healthcare,^3^ socioeconomic inequalities,^3–8^ environmental risks, and geographic disparities.^9–11^ These determinants exhibit considerable spatial variation, resulting in clusters of elevated child mortality that are often masked when only national-level averages are reported. Effective reduction of these disparities requires context-specific interventions that are guided by reliable and detailed data.

 Geographic Information Systems (GIS) integrate health, environmental, and socioeconomic data to reveal spatial and temporal patterns in child mortality and to illuminate the underlying drivers of inequities.^12–14^ Incorporating spatial analysis into health decision-making enables the precise allocation of resources, strengthens service delivery, and extends the reach of preventive interventions, particularly in underserved populations.^15–17^ In practice, these capacities enable public health programs to deploy immunization teams to remote communities, prioritize infrastructure development in high-risk districts, and monitor the effectiveness of interventions in near real-time.^18–20^ The application of spatial analysis must be tailored to the primary drivers of mortality. For infectious diseases such as RSV, influenza, and SARS-CoV-2, GIS excels at modeling spatiotemporal patterns of transmission. This enables officials to correlate virus spread with vaccination coverage, facilitating outbreak forecasting and prevention. Conversely, for non-communicable diseases and injuries, mortality is largely driven by persistent socioeconomic inequities and environmental factors. Here, GIS must integrate individual-level socioeconomic status (SES) data with spatial analysis to uncover the root causes of disparities.^21,22^

 This limited integration must be understood within the broader context of health data system challenges in LMICs. As documented by the World Health Organization and other global health authorities, these settings often grapple with fragmented health information systems, lack of data interoperability, andinadequate digital infrastructure, fundamental barriers that impede any data-driven technology, including GIS.^23–25^ Consequently, while GIS holds transformative potential, its effective implementation within national health policy frameworks remains constrained by these underlying systemic challenges, thereby restricting its contribution to improving child health outcomes.^26,27^

 This policy brief aims to provide a detailed analysis of existing GIS applications in child mortality research and, more critically, to outline specific policy actions to leverage these powerful tools more effectively for improving child health outcomes and achieving global development targets.

## Analysis

 The analysis for this evidence-based policy brief is derived from a 2024 scoping review that investigated the role and applications of Geographic Information Systems (GIS) in child mortality research.^28^ Supplementary File 1 contains the PRISMA flow diagram, detailed search strategies for databases, and a summary table of the included studies. The review applied the Arksey and O’Malley methodological framework. This framework is a structured guide for organizing and summarizing broad research areas. It also followed the Preferred Reporting Items for Systematic Reviews and Meta-Analyses extension for scoping reviews (PRISMA-ScR) to ensure transparent and reliable reporting. A systematic search was conducted in three major English-language databases: PubMed, Scopus, and Web of Science. The search covered studies published between January 1, 2000 and December 25, 2023. The search strategy included terms related to GIS, pediatric populations, and mortality. It used both Medical Subject Headings (MeSH) and Boolean operators to achieve a comprehensive and inclusive scope. Reference lists and citations of relevant studies were also checked to identify additional sources. Clear inclusion and exclusion criteria were applied to maintain quality and relevance. Eligible studies were original research articles published in peer-reviewed English-language journals that used at least one GIS or spatial analysis technique in child mortality research. Studies were excluded if they were written in a language other than English, if they were review articles, letters to the editor, or research protocols, or if they focused on non-child populations or lacked full text. Two reviewers independently screened the titles and abstracts and then assessed the full texts. Any disagreements were resolved through consensus. Data extraction was guided by a predefined checklist that included 12 key variables. These variables covered study characteristics such as title, first author, year of publication, study location, study population, outcome measures, study period, geographical level of analysis (scale), the aim of the study, key results, type of GIS methodology, geospatial and non-geospatial analytical techniques, and type of GIS application. Through a systematic synthesis and categorization process, the applications of GIS were grouped into three main types: disease mapping and surveillance, contextual and risk factor analysis, and health access and planning. The extracted data were checked again by the reviewers to ensure accuracy.

 The structure of the study is based on the World Health Organization guidelines for policy briefs.^29^ Each section reflects important policy dimensions such as cost considerations, equity implications, and the responsibilities of stakeholders. These themes were identified through analysis of GIS implementation and use. Key challenges included financial constraints, limited access, and the need for regulatory frameworks. This structured approach ensures that the policy recommendations are grounded in empirical evidence and practical feasibility. It provides a comprehensive framework to support decision-makers.

## Key Findings from the Scoping Review

 The systematic scoping review underlying this brief initially identified 5,841 records. After screening, 177 studies met the inclusion criteria. The geographical focus of the evidence was predominantly on single countries (n = 156), with the highest density of studies in East Africa (n = 33), Asia (n = 32), and South America (n = 28). An analysis of the GIS methodologies employed showed a heavy reliance on thematic mapping (92.1%) for basic visualization. The use of more advanced techniques was less common: spatial modeling (57.6%) for identifying risk factors, space-time clustering (37.9%) for detecting outbreaks, and GPS (1.7%) for precise data collection. Critically, web-based GIS platforms (0%) were absent. In terms of application, GIS was used primarily for disease mapping and surveillance (95%). Its use for contextual and risk factor analysis (44.6%) was less frequent, and its application to health access and planning (5.6%) was markedly limited.

## Policy Implications and Recommendations

 To situate our recommendations within an established health systems paradigm, we frame them as a spatial enhancement of the WHO’s building blocks.^30^ This perspective reveals how geospatial technology cross-cuts and strengthens core system functions: Recommendation 1 directly modernizes the Health Information Systems block by infrastructuring spatial data capabilities. Recommendations 2 and 3 operationalize this by enabling location-intelligent Service Delivery and breaking down data silos for interoperability. Concurrently, Recommendation 4 builds a spatially-skilled Health Workforce, and Recommendation 5 grounds Leadership and Governance in community-mapped equity data. Thus, our framework’s distinct value lies in systematically embedding a spatial dimension to target geographic health inequities directly. To this end, the following set of policy recommendations has been developed. These recommendations are summarized in Table 1 and are intended to guide health authorities and stakeholders in making informed, data-driven decisions for child health planning.

**Table 1:** Summary of recommendations

**Recommendation**	**Main Advantages**	**Main Disadvantages**	**Cost and Feasibility of Implementation**	**Equity Considerations**	**Stakeholders’ Responsibilities**
Invest in GIS Infrastructure and Governance	• Ensures a consistent and reliable GIS system through predictable funding. • Embeds GIS formally into national health strategies, ensuring its use is not an ad-hoc decision. • Creates a skilled workforce with a focus on local expertise ("GIS Champions").	• Requires significant upfront capital for hardware, software, and training. • Establishing governance and training programs can take a long time, delaying the immediate benefits. • Institutional inertia and a lack of understanding from policymakers may hinder adoption.	• High initial capital expenditure for technology and training. • Securing funding requires strong political will and reallocating existing budgets. • External funding can help, but internal commitment is key for sustainability.	• Ensures all regions, including underserved ones, have access to the same quality of data and tools. • A clear baseline assessment helps identify regions with the greatest needs, ensuring resources are allocated equitably.	• Ministry of Health: Secure funding, integrate GIS into national health budgets, and establish formal governance. • Policymakers: Reallocate existing budgets and ensure political commitment. • Health Managers: Define KPIs to demonstrate value and ensure the system's continued support. • External Donors: Provide initial investment and support to fill funding gaps.
Foster the Adoption of Advanced and Interactive GIS Technologies	• Enables proactive, rather than reactive, public health interventions. • Allows for precise resource allocation to "hotspots" of child mortality. • Interactive platforms facilitate data sharing and cross-sectoral collaboration.	• Requires specialized skills that may be scarce in many health ministries. • Real-time dashboards and data sharing platforms require robust security protocols to prevent data breaches. • Technical and managerial staff need extensive training to effectively use and interpret dynamic tools.	• Requires investment in specialized software and development of custom dashboards. • Pilot programs can demonstrate the value of the technology, making it easier to secure funding for a wider rollout. • Requires skilled developers and GIS professionals.	• By identifying high-risk areas, resources can be directed to populations that need them most. • Real-time dashboards can highlight disparities in service coverage and outcomes, making them harder to ignore.	• Ministry of Health: Prioritize and deploy platforms for dynamic mapping and predictive analytics. • Technical Staff: Develop and maintain secure, real-time dashboards. • Program Managers: Use the platforms to interpret spatial analytics for strategic planning and resource allocation.
Strengthen Data Infrastructure and Interoperability	• Allows for the integration of environmental, socioeconomic, and health data for a more complete understanding of child health. Breaking down data silos unlocks the full potential of GIS for identifying complex risk factors. • Facilitates data exchange between different ministries and agencies.	• Overcoming institutional and technical barriers between different government bodies can be very difficult. • Integrating diverse datasets, especially with location-based data, requires strict privacy and ethical protocols. • Establishing and enforcing a single standard for data interoperability can be challenging and time-consuming.	• Investment is needed for developing APIs, standardizing data systems, and creating a national geodatabase. • Requires a strong mandate from the Ministry of Health and cooperation from other government agencies. • Pilot projects can demonstrate the value of data integration.	• Integrating socioeconomic and environmental data helps identify root causes of disparities, moving beyond a purely medical approach to health. • Satellite imagery and other nontraditional data can help identify hard-to-reach or informal communities that may be left out of traditional health surveys.	• Ministry of Health: Establish technical protocols and create a dedicated data coordination unit. • Statistical Agencies & Other Ministries: Sign data-sharing agreements and adopt interoperability standards. • Data Coordination Unit: Enforce standards, manage data exchange, and ensure data privacy and ethical use.
Promote Interdisciplinary Collaboration and Capacity Building	• Combines epidemiological knowledge with technical GIS skills for more effective problem-solving. • Mixed expert teams can lead to new and creative approaches to public health challenges. • Formal curricula and workshops ensure knowledge is shared and institutionalized, rather than staying with a few individuals.	• Overcoming professional and departmental silos can be challenging and requires a shift in organizational culture. • Creating new units and funding extensive training programs can be costly and compete with other priorities. • Interdisciplinary collaboration and training take time and effort from all parties, who may already have heavy workloads.	• Involves funding for training programs, workshops, and the establishment of new units. • This is more of a cultural and organizational change than a technical one. It can be implemented incrementally through small, focused teams and pilot projects.	• Ensures that solutions are co-developed, taking into account diverse perspectives and local contexts. • Training health administrators and policymakers in spatial literacy enables them to make more informed decisions that benefit all populations.	• Ministry of Health: Establish mixed expert teams and a dedicated geospatial health unit. • Academic Institutions: Develop specialized curricula and short-course programs. • Health Professionals, GIS Specialists, Data Scientists: Participate in joint workshops and mixed teams to co-develop solutions.
Enhance Community Engagement and Equity-Focused Spatial Planning	• Incorporates community knowledge to ensure interventions are culturally appropriate and effective. • Community-collected data can fill gaps missed by national systems, providing a more accurate picture of local assets and risks. • When communities are involved in the planning, they are more likely to accept and sustain the interventions.	• Community-collected data may be inconsistent or lack the rigor of formal surveys. • Participatory mapping and community training require significant time and effort. • If not managed properly, community-level data collection could be influenced by local politics or power dynamics.	• Involves training community health workers and developing simple mobile tools. • Can be implemented through pilot programs in specific communities to demonstrate value before a wider rollout. • Requires a strong emphasis on trust-building with local populations.	• Ensures that health planning is not just about geography but also addresses the unique needs of different income levels, ethnic groups, and urban/rural populations. • Makes local inequalities visible and actionable, holding health managers accountable for addressing them.	• Ministry of Health: Promote and fund participatory mapping initiatives. • Health Managers: Integrate community data into district and national systems. • Community Health Workers & Local Communities: Collect hyper-local data and participate in planning processes.

###  Recommendation 1: Invest in GIS Infrastructure and Governance for Health System Planning

 Integrating GIS into health planning requires sustained financial and institutional commitment. Evidence from multiple health systems indicates that insufficient funding for hardware, software, and spatial data acquisition is a significant barrier to the sustained use of GIS. Policymakers should secure predictable funding for GIS by reallocating a portion of existing health infrastructure budgets, for example, by diverting part of construction or equipment resources toward geospatial planning, while also seeking external donor or partner support to fill initial investment gaps. To guarantee long-term sustainability, GIS must be included as a routine line item in national and subnational health budgets, ensuring that system maintenance, data updates, and technical support receive consistent financial backing. Beyond infrastructure, investment in human capital is essential. GIS training should be integrated into public health curricula, staff certification programs should be established, and dedicated “GIS Champion” roles created at subnational levels to sustain local expertise. GIS must be formally embedded in national health strategies, supported by clear governance mechanisms, role assignments, and rigorous data privacy protocols to ensure ethical use. To demonstrate value and secure continued political commitment, Key Performance Indicators (KPIs) should be defined, such as reduced travel time to health facilities, improved emergency response times, or increased vaccination coverage in underserved clusters. As an immediate first step, a rapid technical capacity and data-gap assessment within the Ministry of Health should be commissioned to establish a clear baseline for budgetary planning and governance design

###  Recommendation 2: Foster the Adoption of Advanced and Interactive GIS Technologies

 Current applications of GIS in public health planning remain largely reliant on static maps and retrospective analysis, which severely limit their utility for proactive intervention and real-time resource allocation. To address this gap, policy should actively promote a shift towards dynamic, interactive, and predictive spatial technologies. Policymakers should prioritize deploying GIS platforms capable of dynamic mapping, scenario modeling, and predictive analytics to support proactive public health interventions. For infectious disease mortality, this means developing models that can point out areas where vaccination efforts need to be intensified upon detecting early signals of an upcoming outbreak (e.g., of RSV or influenza). These platforms are essential for integrating disparate data sources, enabling cross-sectoral collaboration, and providing actionable intelligence to field workers and managers. Pilot programs in high-risk regions can demonstrate the operational value of web-based GIS applications for monitoring child mortality and optimizing resource allocation. Secure, real-time dashboards should be developed to provide spatially explicit indicators directly to health managers, enabling the rapid identification of emerging mortality hotspots and the continuous monitoring of intervention effectiveness. Targeted training programs are essential for both technical staff and program managers, emphasizing the interpretation of spatial analytics for strategic planning, emergency response, and evidence-based resource allocation. Measurable outcomes and key performance indicators, such as reduced response times to emergencies, improved targeting of interventions, or increased coverage of health services in high-risk areas, should be defined to ensure that GIS adoption translates into tangible improvements in child health outcomes. By combining technology deployment with focused capacity building, GIS can evolve into an actionable decision-support system that enables timely, data-driven, and spatially informed public health actions.

###  Recommendation 3: Strengthen Data Infrastructure and Interoperability for Spatial Analysis

 Effective spatial analysis requires integrating diverse data sources beyond health statistics, including environmental, socioeconomic, and infrastructure data. Currently, siloed data systems and inconsistent standards impede this integration, limiting the analytical power of GIS in public health decision-making. Health ministries should establish technical protocols for spatial data interoperability, mandating standardized coordinate systems, metadata requirements, and common geospatial identifiers across all health information systems. This requires integrating spatial data standards into existing health data platforms and developing application programming interfaces (APIs) to enable secure data exchange with statistical agencies, environmental departments, and infrastructure ministries. Priority should be given to creating a national geodatabase for child health indicators, incorporating both facility-based reporting and population-based surveys. This infrastructure must include robust data governance frameworks that address privacy, security, and the ethical use of location-based health data. Pilot projects should demonstrate the integration of nontraditional data sources, such as satellite imagery, to monitor air quality, land use patterns, vegetation cover, and water resources as part of environmental exposure assessment with routine health statistics to identify novel risk factors and optimize resource allocation. Alongside data integration, a specific focus on improving the quality of cause-of-death data is needed. This can be achieved by sponsoring quality assurance studies that validate cause-of-death records against medical records and by integrating standardized verbal autopsy methods into national health information systems. Implementation requires appointing a dedicated data coordination unit within the health ministry. This unit should be mandated not only to enforce interoperability standards and facilitate cross-sectoral data sharing but also to oversee data quality assurance initiatives, including the validation of cause-of-death data.

###  Recommendation 4: Promote Interdisciplinary Collaboration and Capacity Building

 The effective application of GIS in public health requires integration of diverse expertise beyond traditional health sector capabilities. Current operational silos between health professionals, GIS specialists, data scientists, and environmental researchers limit the potential for innovative spatial analysis and effective implementation. Health ministries should establish formal mechanisms for interdisciplinary collaboration by creating mixed expert teams at national and regional levels. These teams should co-develop spatial analyses and intervention plans, combining epidemiological insight with technical geospatial expertise. Specifically, the formation of a dedicated geospatial health unit within the national public health institute would provide institutional anchoring for this integrated approach. Academic institutions must be engaged to develop specialized training curricula that bridge public health and spatial sciences. This includes integrating GIS and spatial analysis core competencies into public health curricula, while simultaneously developing short-course certification programs for current health workforce members. Particular emphasis should be placed on practical skills for interpreting and applying spatial data in public health decision-making rather than technical software proficiency. Capacity building should extend beyond technical staff to include health administrators and policymakers, focusing on spatial literacy and interpretation of geospatial analytics for resource allocation decisions. Regular knowledge-sharing forums and joint problem-solving workshops between health departments, statistical agencies, and academic institutions will foster sustained collaboration and continuous skill development.

###  Recommendation 5: Enhance Community Engagement and Equity-Focused Spatial Planning

 While GIS is highly effective at identifying geographical disparities, relying only on centrally driven GIS analysis without local participation often overlooks the community-specific barriers and sociocultural factors that sustain high child mortality. To ensure that spatial planning leads to effective and sustainable interventions, community engagement must be systematically integrated into GIS-based decision-making. Policymakers should promote participatory mapping initiatives, training community health workers to collect hyper-local data using simple mobile GIS tools. This approach allows communities to map their own assets, such as water sources and informal health providers, as well as risks like unsafe roads or missing clinics, capturing data that national systems may miss. National child mortality dashboards, as suggested in earlier recommendations, should include indicators that focus on equity, showing disparities not only between regions but also within each region across income levels, ethnic groups, and urban-rural areas. This makes inequalities visible and actionable for health managers. Community-collected data should be formally integrated into district and national health information systems, so that local priorities directly guide planning and resource allocation. By incorporating community knowledge into spatial analysis and focusing on equity, health systems can design interventions that target the right areas, fit local conditions, respect cultural norms, and are more likely to be accepted and adopted by the people they aim to serve. To ensure the reliability and sustainability of this approach, robust data quality assurance mechanisms must be integrated from the outset. This includes developing simple standardized digital forms with built-in validation checks, providing ongoing training and supervision for community health workers, establishing regular data auditing procedures, and creating feedback loops where communities can verify and contextualize the collected data.

## Considerations for Implementation

 Successful implementation of the proposed GIS framework requires addressing critical operational challenges beyond technical infrastructure. Evidence from digital health initiatives in LMICs highlights several pivotal considerations for sustainable adoption.

###  Sustaining Political Commitment and Financial Viability

 Building and maintaining political will beyond initial donor funding requires strategic approaches. Research shows that incorporating GIS operational costs into routine government health budgets greatly improves sustainability.^31^ Additionally, demonstrating concrete returns on investment, such as lowered transportation costs through optimized vaccination routes or enhanced resource allocation efficiency, can help secure ongoing government support.^19^ Establishing multi-sectoral steering committees with representation from finance and planning ministries has proven effective in maintaining high-level commitment to geospatial health initiatives.^32^

###  Ensuring Frontline Adoption and Usability

 The risk of technological solutions failing due to a lack of health worker buy-in is well-documented. Studies show that involving frontline workers in GIS tool design significantly improves adoption rates.^33^ Effective strategies include developing user-centered interfaces that minimize data entry burden, providing continuous contextual training rather than one-time technical sessions, and aligning digital tools with existing workflow patterns.^34^ Successful programs often designate “on-site GIS specialists” among health staff to facilitate peer-to-peer support and troubleshoot implementation challenges.^35^

###  Balancing Data Governance and Interoperability

 The tension between robust data privacy and real-time data sharing requires careful navigation. Implementation should adhere to privacy-by-design principles, incorporating data anonymization and aggregation protocols from the outset.^36^ Developing tiered data access frameworks that distinguish between operational data for frontline workers and sensitive individual-level data for authorized analysts can reconcile these competing needs.^37^ Establishing clear data governance policies that define ownership, access rights, and security protocols is essential for maintaining trust while enabling necessary data sharing.^38^

###  Data Relevance and Timeliness

 The value of a GIS is contingent on the quality and currency of its underlying data. Implementation must include robust mechanisms for continuous data collection and validation, particularly at the community level. This can be achieved by integrating GIS data streams with routine health information systems and investing in rapid, mobile-based data capture tools for community health workers to ensure that spatial insights reflect on-the-ground realities without significant time lag.^39,40^

###  Planning for Scalability and Replicability

 Pilot projects must be designed with scale in mind from the outset. Successful implementation requires a phased approach, starting in high-priority regions with clear metrics for success, before expanding nationally. Developing reusable, standardized data models and toolkits can enable successful approaches to be efficiently replicated across different districts or countries, maximizing the return on initial investments.^41^

 Looking beyond its immediate relevance to child mortality, the policy framework proposed in this brief contributes to the broader digital health transformation agenda in LMICs. By embedding spatial intelligence within routine health information systems, it operationalizes key priorities of the WHO Global Strategy on Digital Health, including interoperability, data governance, and evidence-driven planning.^23^ This integration positions GIS not as a stand-alone innovation but as an enabling layer that enhances the functionality and responsiveness of digital health ecosystems. In doing so, it bridges the gap between traditional health information systems, often limited to facility-based reporting, and the next generation of connected, context-aware platforms capable of integrating community, environmental, and demographic data.^42^ Thus, the proposed actions advance the global discourse on how LMICs can transition from fragmented data infrastructures toward intelligent, spatially enabled health systems that support equitable and data-driven decision-making.^43^

## Conclusion

 The integration of Geographic Information Systems into child health surveillance and planning offers a transformative opportunity to reduce under-five mortality. Moving beyond static mapping and fragmented data use, the recommendations presented in this policy brief outline a comprehensive pathway for building sustainable GIS infrastructure, strengthening data systems, and embedding geospatial analysis into routine decision-making. Together, these actions can enable health authorities to detect mortality hotspots in real time, allocate resources more efficiently, and design interventions that are contextually appropriate and equitable.

 Achieving this transformation requires coordinated commitment from multiple stakeholders, including ministries of health, statistical agencies, academic institutions, and local communities. Investment in infrastructure must be paired with data interoperability, capacity building, and meaningful community participation to ensure that spatial insights translate into tangible health gains. When effectively implemented, these strategies will shift GIS from a descriptive tool to a dynamic decision-support system, supporting proactive interventions, enhancing health equity, and ultimately saving children’s lives.

## Competing Interests

 The authors declare that they have no competing interests.

## Ethical Approval

 Not applicable.

## Supplementary Files

Supplementary file 1 contains the PRISMA flow diagram, detailed search strategies for the databases, and a summary table of the included studies.
