# Supplementary material for: Utilizing GIS to Reduce Child Mortality: Strategic Policy Actions for Health Equity
Source: Health Promot Perspect. 2026 Aug 21;16(2):201–8. doi: 10.34172/hpp.45251 (PMC13723422; doi:10.34172/hpp.45251)
Supplement: Supplementary file 1 [file hpp-16-201-s001.pdf]

Samimi et al, Health Promotion Perspectives 2026;16(2):201-S1
doi:10.34172/hpp.45251
https://hpp.tbzmed.ac.ir

1. Detailed search strategies for all databases:

Data base	Search date	Number of studies
**PubMed**	**12/25/2023**	**1885**
**Scopus**	**12/25/2023**	**2380**
**Web of science**	**12/25/2023**	**1576**

2. Search strategy in PubMed:

((((((((((((((((((((((((((((((((((((((geographical distribution[Title/Abstract]) OR (geographic distribution[Title/Abstract])) OR (spatial analysis[Title/Abstract])) OR (spatial analysis[MeSH Major Topic])) OR (geospatial analysis[Title/Abstract])) OR (geographic mapping[Title/Abstract])) OR (geographic mapping[MeSH Major Topic])) OR (geographic information system[MeSH Major Topic])) OR (geographic information system[Title/Abstract])) OR (geography information system[Title/Abstract])) OR (geographical information system[Title/Abstract])) OR (geographical mapping[Title/Abstract])) OR (Spatio-Temporal Analysis[Title/Abstract])) OR (Spatio-Temporal Analysis[MeSH Major Topic])) OR (Spatio Temporal*[Title/Abstract])) OR (Spatial*[Title/Abstract])) OR (Spatiotemporal*[Title/Abstract])) OR (Georeferenc*[Title/Abstract])) OR (Mapping[Title/Abstract])) OR (GIS[Title/Abstract])) OR (Hotspot analys*[Title/Abstract])) OR (geospatial*[Title/Abstract])) OR (Cluster analysis[Title/Abstract])) OR (Cluster analysis[MeSH Major Topic])) OR (choropleth map*[Title/Abstract])) OR (heat map*[Title/Abstract])) OR (dasymetric map*[Title/Abstract])) OR (Spatial Autocorrelation[Title/Abstract])) OR (geographic cartography[Title/Abstract])) OR (spatial regression[Title/Abstract])) OR (spatial regression[MeSH Major Topic])) OR (Spatial inequality[Title/Abstract])) OR (Geographical inequality[Title/Abstract])) OR (Spatial Dependency[Title/Abstract])) OR (Geographic Dependency[Title/Abstract])) OR (Geographically Weighted Regression[Title/Abstract])) OR (Space-Time Geography[Title/Abstract])) AND (((((((((((((((((("under 5 years old"[Title/Abstract]) OR ("under 5 years"[Title/Abstract])) OR (child[Title/Abstract])) OR (child[MeSH Major Topic])) OR (Neonatal[MeSH Major Topic])) OR (Neonatal[Title/Abstract])) OR (children[Title/Abstract])) OR (infant[Title/Abstract])) OR (infant[MeSH Major Topic])) OR (fetal[MeSH Major Topic])) OR (fetal[Title/Abstract])) OR (perinatal[Title/Abstract])) OR (Newborn*[Title/Abstract])) OR (Newborn[Title/Abstract])) OR (perinatal[MeSH Major Topic])) OR (neonate[Title/Abstract])) OR ("Children Under Five"[Title/Abstract]))) AND ((mortality[Title/Abstract]) OR (mortality[MeSH Major Topic]))

3. **PRISMA flowchart of study selection:**

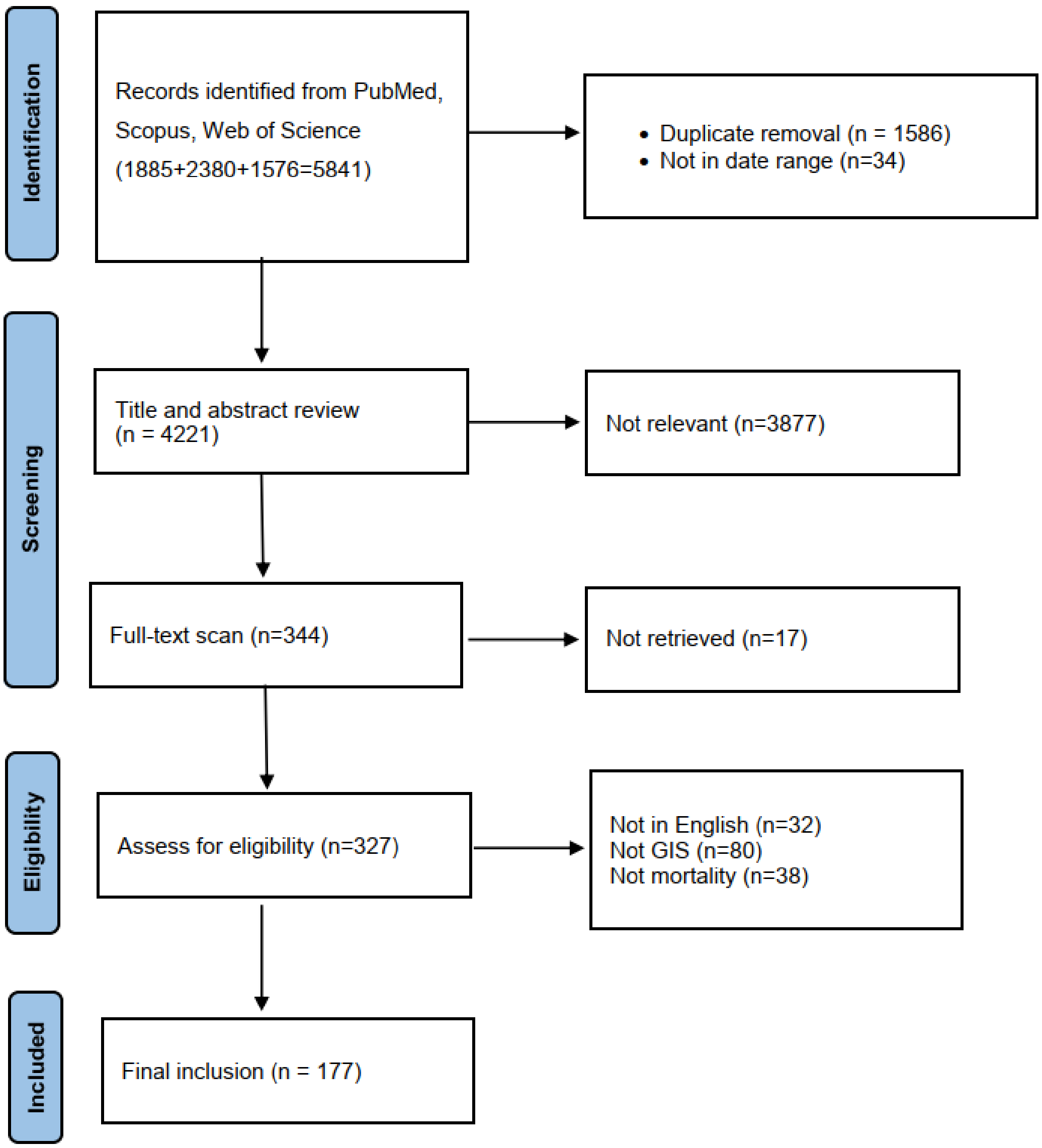

4. A summary table aggregating key characteristics of all included studies (n=177).

Characteristic	Category	Number of Studies (%)
**Geographical Focus**	Single-Country	156 (88.1%)
	Multi-Country	16 (9.0%)
	Global	5 (2.8%)
**GIS Methodology**	Thematic Mapping	163 (92.1%)
	Spatial Modeling	102 (57.6%)
	Space-Time Clustering	67 (37.9%)
	GIS/GPS Tools	3 (1.7%)
	Web-GIS	0 (0%)
**GIS Application**	Disease Mapping & Surveillance	168 (95.0%)
	Contextual & Risk Factor Analysis	79 (44.6%)
	Health Access & Planning	10 (5.6%)
